# Solarplast^®^—An Enzymatically Treated Spinach Extract

**DOI:** 10.3390/plants12142678

**Published:** 2023-07-18

**Authors:** Annie Simon, Shahneela Mazhar, Ekaterina Khokhlova, Natasha Leeuwendaal, Christopher Phipps, John Deaton, Kieran Rea, Joan Colom

**Affiliations:** 1 ADM Cork H&W Limited, Food Science Building, University College Cork, T12 Y337 Cork, Ireland; 2 ADM Deerland Probiotics and Enzymes, 3800 Cobb International Boulevard, Kennesaw, GA 30152, USA

**Keywords:** Solarplast^®^, spinach, antioxidant, oxidative stress, HT-29, HepG2

## Abstract

In the modern world we are constantly bombarded by environmental and natural stimuli that can result in oxidative stress. Antioxidant molecules and enzymes help the human body scavenge reactive oxygen species and prevent oxidative damage. Most organisms possess intrinsic antioxidant activity, but also benefit from the consumption of antioxidants from their diet. Leafy green vegetables such as spinach are a well-researched rich source of dietary antioxidant molecules. However, plant cell walls are difficult to digest for many individuals and the bio-accessibility of nutrients and antioxidants from these sources can be limited by the degree of digestion and assimilation. Through a specific enzymatic process, Solarplast^®^ contains organic spinach protoplasts without the cell wall, which may facilitate higher yield and efficacy of beneficial antioxidant molecules. In this study, analytical techniques coupled to in vitro bioassays were used to determine the potential antioxidant activity of Solarplast^®^ and determine its antioxidant enzymatic capabilities. Solarplast^®^ demonstrated superior antioxidant activity when compared to frozen spinach leaves in TOC, FRAP and TEAC antioxidant assays. Several antioxidant enzymes were also increased in Solarplast^®^, when compared to frozen spinach. As a functional readout, Solarplast^®^ attenuated hydrogen peroxide-, ethanol- and acetaminophen-induced increases in oxidative stress and cytotoxicity in both intestinal (HT-29) and liver (HepG2) cell lines. These findings suggest that Solarplast^®^ may represent a non-GMO, plant-based food supplement to help reduce oxidative stress in the human body.

## 1. Introduction

Antioxidants are defined as molecules that can prevent the oxidation of a substrate at low concentrations [1]. These molecules have an important role in the human body by scavenging reactive oxygen species (ROS) and preventing oxidative stress [2]. Oxidative stress occurs when there is an imbalance of the redox reactions in an organism, with an increased presence of ROS compared to antioxidants. The overproduction of such molecules and radicals (superoxide, hydroxyl radical and hydrogen peroxide) can cause oxidative damage to lipids, proteins and DNA [3,4]. This can ultimately lead to cell death, or contribute to the aging process of the body or the development of several diseases including cancer, neurological and digestive disorders [5]. Our bodies normally regulate oxidative stress through natural endogenous and dietary antioxidants, but the efficiency of this protective physiological process can decrease as we age, or in times of stress or illness. The majority of the antioxidants in food are found in plants and are known as phytochemicals [2].

Spinach (*Spinacia oleracea* L.) is widely regarded as a functional food due to its diverse nutritional composition, including chlorophyll, carotenoids, ascorbate (AsA) and dehydroascorbate (DHA), reduced glutathione (GSH) and oxidised glutathione (GSSG), beta-carotene, lutein and various flavonoids that also function as antioxidants [6,7,8]. It contains several essential amino acids, vitamins (vitamin C, vitamin A and vitamin E), minerals (magnesium, manganese, iron and calcium) and folic acid, with a cup of spinach leaves (25 g) providing recommended daily intake doses of vitamin K (15.1%), vitamin A (16%), vitamin C (17.5%) and folic acid (24.5%) for an adult human [9]. Antioxidant enzymes present in spinach include superoxide dismutase (SOD), peroxidase (POD), catalase (CAT), ascorbate peroxidase (APX), glutathione peroxidase (GPX), glutathione S-transferase (GST), monodehydroascorbate reductase (MDHAR), dehydroascorbate reductase (DHAR) and glutathione reductase (GR) [7]. The presence of all these proteins and molecules make spinach a good potential source of antioxidants to help control levels of ROS in the human body. Several studies have employed a combination of Trolox equivalence antioxidant capacity (TEAC) and ferric-reducing antioxidant power (FRAP) assays to evaluate the antioxidant activity of green and dark leafy vegetables including spinach [10,11,12,13]. Pellegrini et al. employed FRAP and TEAC tests to evaluate the total antioxidant capacity of a variety of foods commonly consumed in Italy, including 34 vegetables, out of which spinach had the highest antioxidant capacity [14]. 

In several in vivo models, spinach extracts were effective at preventing lipid peroxidation in skin and metabolic damage in kidney and liver [15,16,17]. Additionally, there is some evidence of spinach and its extracts to have anticancer and antioxidant effect in hepatocellular carcinoma cells (HepG2) and human colon carcinoma cells (HT-29) cell models [6,18,19]. Spinach also alleviated oxidative stress and muscle damage in well-trained healthy young men [20]. However, to be effective, 20,000 g of spinach had to be homogenised, concentrated and purified by chromatography or else ingested in high amounts (1 g/kg body weight) [20,21]. This implies that antioxidant molecules in spinach may be highly diluted by its water content and difficult to access because of plant cell wall resistance to the native enzymes present in the human gastrointestinal tract [22]. Furthermore, as omnivores, we do not have the same innate enzymatic efficiency as herbivores to digest, assimilate and absorb nutrients and antioxidants from plant-based sources. For this reason, most plant foods must be processed (e.g., boiling or cooking) to ensure easier digestion and higher nutrient absorption. However, such processing methods influence the antioxidant content, bioavailability and activity of micronutrients present in plant-based foods [23]. An alternative to all the above is enzymatic digestion of plant cell walls forming protoplasts. This technique has been used for many years on multiple plant species showing consistent and reproducible results [24,25,26]. Solarplast^®^, a novel, commercially available extract from non-GMO, blanched frozen spinach uses enzymatic digestion to remove spinach cell walls, delivering concentrated spinach protoplasts instead of whole spinach cells. The lack of a cell wall facilitates ready access to the rich source of antioxidant molecules and enzymes inherent in spinach. The process is ended by freeze drying the resultant spinach digest to produce a lyophilised spinach protoplast extract.

In this study, we compared the total antioxidant capacity, which is the cumulative capacity of a formulation to scavenge free radicals, using a combination of TOC, FRAP and TEAC assays, along with enzymatic assays (SOD, CAT, POD, GST, MDHAR and GPx) of Solarplast^®^ with blanched frozen spinach leaves. Additionally, as a functional readout, we assessed the ability of Solarplast^®^ to reduce oxidative stress induced by oxidants such as hydrogen peroxide, ethanol and acetaminophen on the HT29, a cell line representative of the gastrointestinal tract and human liver carcinoma cell line HepG2 and compared with a powerful antioxidant, vitamin C. 

## 2. Results

### 2.1. Total Antioxidant Capacity of Solarplast^®^ and Frozen Spinach

**Figure 1:**
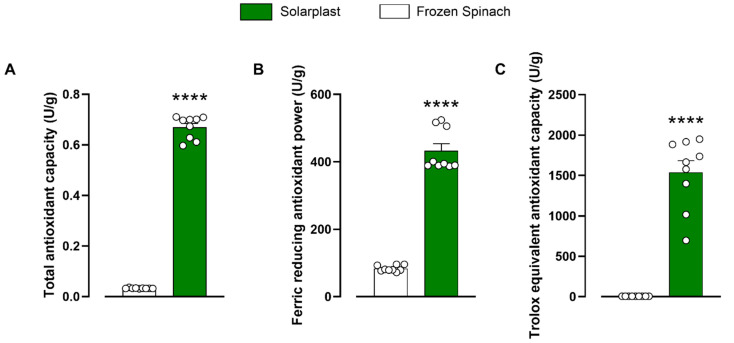
Solarplast^®^ had significantly higher total antioxidant activity than frozen spinach extracts. Total antioxidant activity was measured using (**A**) Trolox (TOC), (**B**) ferric reducing (FRAP) and (**C**) total antioxidant capacity (TEAC). Bars indicate average concentration (n = 9) ± SEM. Significantly higher than frozen spinach, **** *p* < 0.0001.

To fully assess the presence of small antioxidant molecules and antioxidant enzymes, 50 mg/mL extracts of Solarplast^®^ and organic frozen spinach were characterised in three different total antioxidant capacity assays. Solarplast^®^ demonstrated superior antioxidant capacity as compared with frozen spinach for total antioxidant capacity (Figure 1A, t(16) = 42.37, *p* < 0.0001), ferric-reducing antioxidant power (Figure 1B, t(16) = 16.68, *p* < 0.0001) and Trolox equivalent antioxidant capacity (Figure 1C, t(16) = 10.65, *p* < 0.0001).

### 2.2. Solarplast^®^ and Frozen Spinach Individual Antioxidant Activity

**Figure 2:**
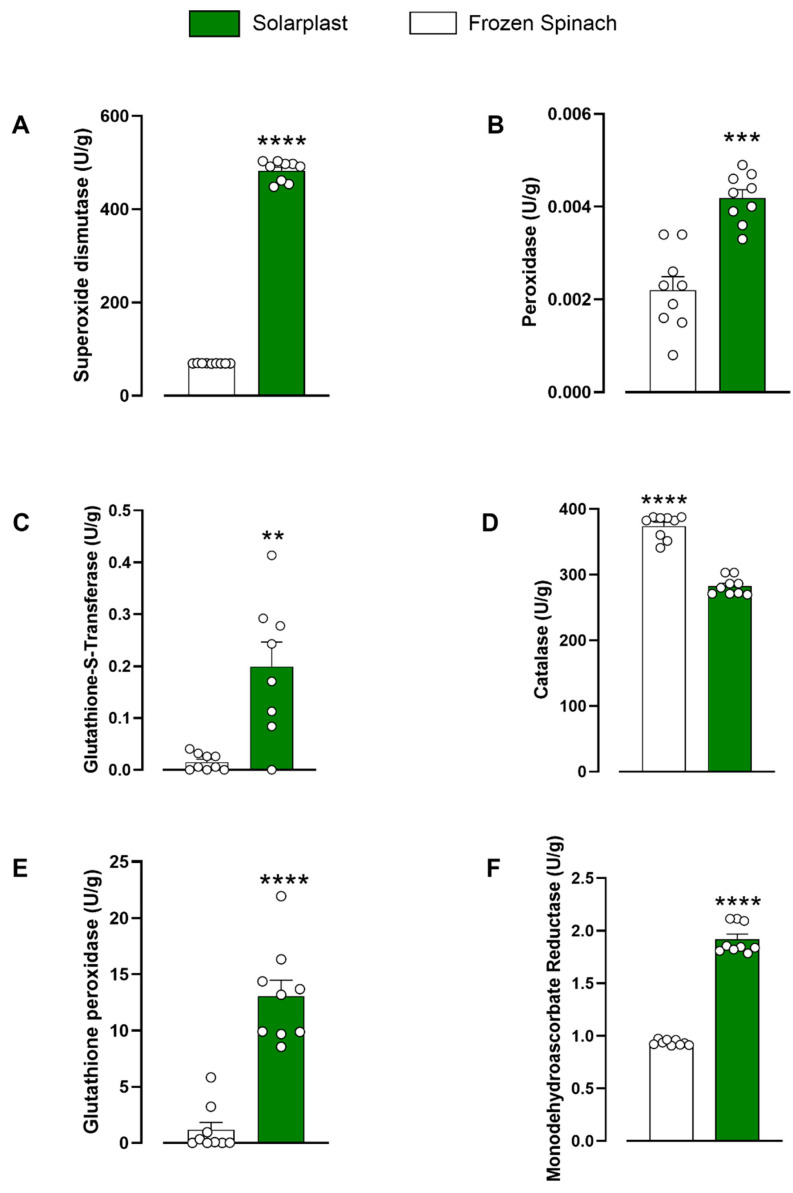
Solarplast^®^ had significantly higher levels of antioxidant enzymes compared to frozen spinach except for catalase. (**A**) Superoxide dismutase, (**B**) peroxidase, (**C**) glutathione-S-transferase, (**D**) catalase, (**E**) glutathione peroxidase and (**F**) monodehydroascorbate reductase. Results show average (n = 6) ± SEM. ** *p* < 0.01, *** *p* < 0.001 and **** *p* < 0.0001 indicate significantly higher concentrations.

The activity of seven antioxidant enzymes was tested in 50 mg/mL Solarplast^®^ and frozen spinach extracts. Solarplast^®^ demonstrated superior enzymatic activity over frozen spinach for superoxide dismutase (Figure 2A, t(16) = 56.49, *p* < 0.0001), peroxidase (Figure 2B, t(16) = 5.890, *p* < 0.001), glutathione-S-transferase (Figure 2C, Mann–Whitney test U = 7.5, *p* < 0.01), glutathione peroxidase (Figure 2E, t(13) = 5.925, *p* < 0.0001) and monodehydroascorbate reductase (Figure 2F, t(16) = 20.41, *p* < 0.0001). Conversely, catalase concentration was higher in frozen spinach 374 ± 6.0 U/g than in Solarplast^®^ 282.5 ± 4.5 U/g (Figure 2D, t(16) = 12.17, *p* < 0.0001).

### 2.3. Solarplast^®^ Safety in HT-29 and HepG2 Cell Lines

**Figure 3:**
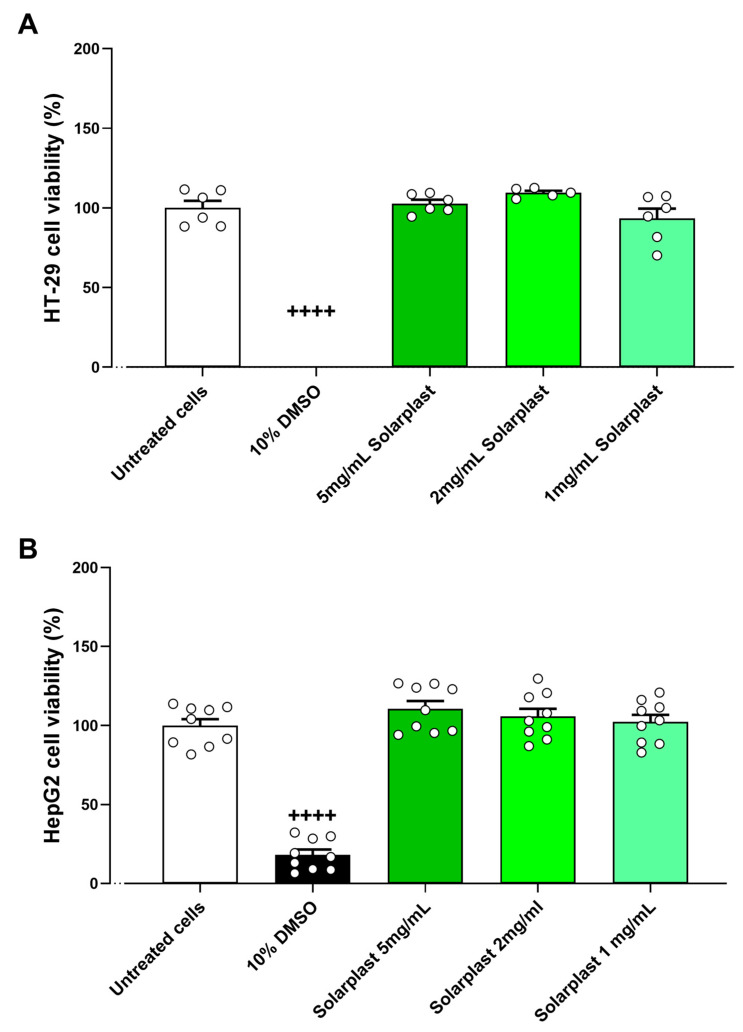
Solarplast^®^ extracts were not cytotoxic in intestinal HT-29 and liver HepG2 cell lines. (**A**) Solarplast^®^ was not cytotoxic in HT-29 cell line. Results show average survival (n = 6) ± SEM. (**B**) Solarplast^®^ was not cytotoxic in HepG2 cell line. Results show average survival (n = 9) ± SEM. ++++ *p* < 0.0001 significantly higher than untreated cells.

After determining Solarplast^®^ increased antioxidant activity, its extracts were tested for cytotoxic effects and capacity to protect against oxidative stress on intestinal and liver cell lines. The positive control 10% DMSO significantly reduced cell viability compared to untreated cells in HT-29 (Figure 3A, t(10) = 25.16, *p* < 0.0001) and HepG2 (Figure 3B, t(16) = 15.41, *p* < 0.0001). There were no cytotoxic effects of Solarplast^®^ at any concentration for HT-29 (Figure 3A, F(3, 24) = 2.082, *p* = 0.1486) or Hep2G (Figure 3B, F(3, 24) = 1.076, *p* = 0.3781). 

### 2.4. Solarplast^®^ Antioxidant Capacity in HT-29 and HepG2 Cell Lines

**Figure 4:**
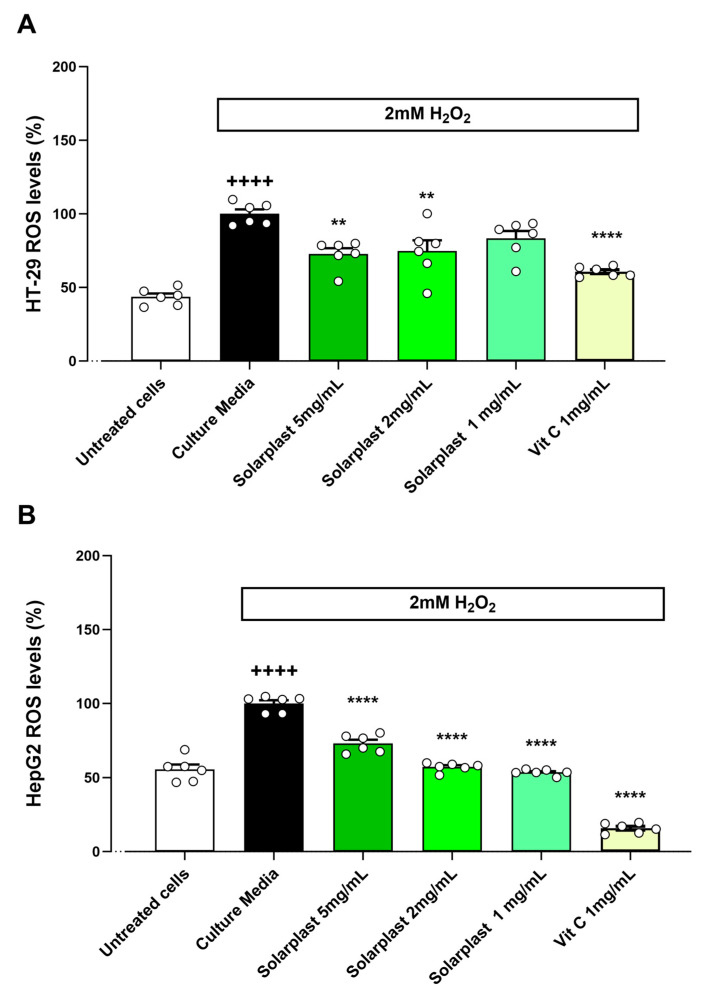
Solarplast^®^ extracts reduced ROS levels in intestinal HT-29 and liver HepG2 cell lines exposed to H_2_O_2_. (**A**) Solarplast^®^ reduced ROS concentration in HT-29 cells exposed to H_2_O_2_. Results show average ROS production (n = 6) ± SEM. (**B**) Solarplast^®^ reduced ROS concentration in HepG2 cells exposed to H_2_O_2_ (control). Results show average ROS production (n = 6) ± SEM. All cells were exposed to 2 mM H_2_O_2_ except for the untreated group. ** *p* < 0.01 and **** *p* < 0.0001 indicate significantly lower than control H_2_O_2_-exposed cells. ++++ *p* < 0.0001 significantly higher than untreated cells.

Hydrogen peroxide increased ROS levels as compared to untreated cells for HT-29 (Figure 4A, t(10) = 14.80, *p* < 0.001) and HepG2 (Figure 4B, t(10) = 11.18, *p* < 0.001) cell lines. Solarplast^®^ significantly attenuated this hydrogen-peroxide-induced increase in ROS production in both HT-29 (Figure 4A, F(4, 20) = 13.82, *p* < 0.0001) and HepG2 (Figure 4A, F(4, 20) = 396.7, *p* < 0.0001) cell lines.

**Figure 5:**
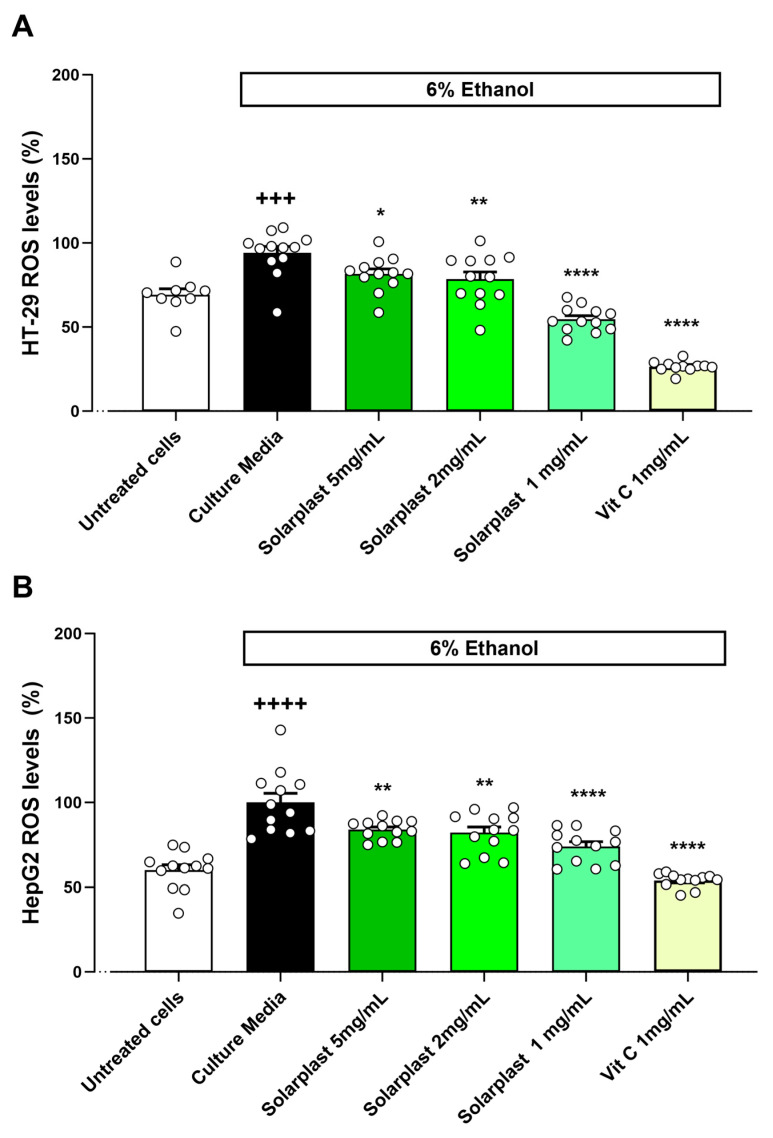
Solarplast^®^ extracts reduced ROS levels in intestinal HT-29 and liver HepG2 cell lines exposed to 6% ethanol. (**A**) Solarplast^®^ reduced ROS concentration in HT-29 cells exposed to ethanol. Results show average ROS production (n = 12) ± SEM. (**B**) Solarplast^®^ reduced ROS concentration in HepG2 cells exposed to ethanol. Results show average ROS production (n = 12) ± SEM. All cells were exposed to 6% ethanol except for the untreated group. * *p* < 0.05, ** *p* < 0.01 and **** *p* < 0.0001 indicate significantly lower than control ethanol-exposed cells. +++ *p* < 0.001 and ++++ *p* < 0.0001 significantly higher than untreated cells.

Ethanol also increased ROS levels as compared to untreated cells for HT-29 (Figure 5A, t(19) = 4.585, *p* = 0.002) and HepG2 (Figure 5B, t(22) = 6.312, *p* < 0.001) cell lines. Solarplast^®^ significantly attenuated this ethanol-induced increase in ROS production in both HT-29 (Figure 5A, F(4, 43) = 61.35, *p* < 0.0001) and HepG2 (Figure 5A, F(4, 44) = 27.41, *p* < 0.0001) cell lines.

**Figure 6:**
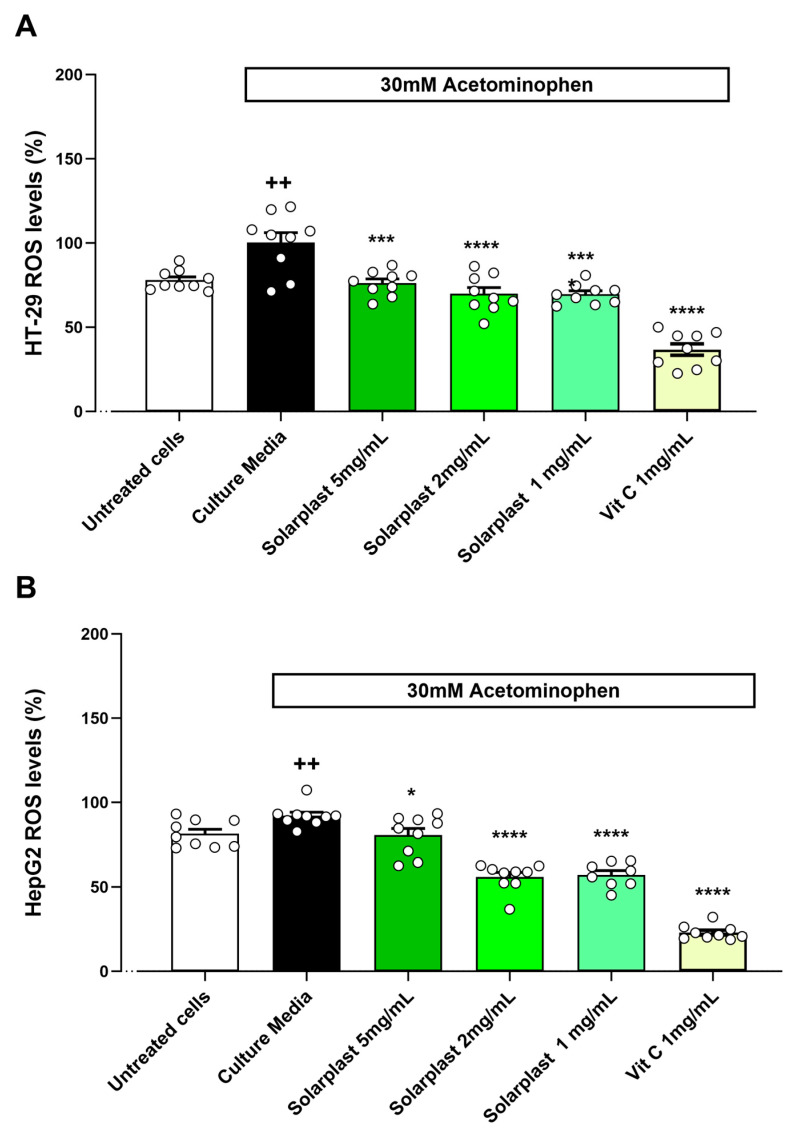
Solarplast^®^ extracts reduced ROS levels in intestinal HT-29 and liver HepG2 cell lines exposed to acetaminophen. (**A**) Solarplast^®^ reduced ROS concentration in HT-29 cells exposed to acetaminophen. Results show average ROS production (n = 9) ± SEM. (**B**) Solarplast^®^ reduced ROS concentration in HepG2 cells exposed to acetaminophen. Results show average ROS production (n = 9) ± SEM. All cells were exposed to 30 mM acetaminophen except for the untreated group. * *p* < 0.05, *** *p* < 0.0005 and **** *p* < 0.0001 indicate significantly lower than control acetaminophen-exposed cells. ++ *p* < 0.01 significantly higher than untreated cells.

Similarly, acetaminophen increased ROS levels as compared to untreated cells for HT-29 (Figure 6A, t(16) = 3.582, *p* = 0.0025) and HepG2 (Figure 6B, t(16) = 3.075, *p* = 0.0072) cell lines. Solarplast^®^ significantly attenuated this acetaminophen-induced increase in ROS production in both HT-29 (Figure 6A, F(4, 29) = 28.62, *p* < 0.0001) and HepG2 (Figure 6A, F(4, 28) = 102.4, *p* < 0.0001) cell lines.

## 3. Materials and Methods

### 3.1. Materials

Solarplast^®^ was obtained from ADM Deerland Probiotic & Enzymes, Kennesaw Georgia, USA. Solarplast^®^ is prepared from non-GMO, blanched frozen spinach using enzymatic digestion to remove spinach cell walls, followed by freeze drying to produce a lyophilised spinach protoplast extract.

Blanched frozen spinach (SuperValu, Cork, Ireland) was purchased from SuperValu, Ireland. Liquid nitrogen was purchased from Irish oxygen ltd, Ireland. Phosphate buffer saline 50X (PBS), minimum eagle medium (MEM), Dulbecco’s Modified Eagle’s Medium—high glucose (DMEM), fetal bovine serum, non-essential amino acids, penicillin, streptomycin and L-Glutamine were obtained from Capricorn Scientific, Ebsdorfergrund, Germany. A superoxide dismutase (SOD) assay kit, peroxidase (POD) assay kit and total antioxidant capacity (TOC) assay kit were obtained from Sigma-Aldrich, St. Louis, MO, USA. Catalase (CAT) and monodehydroascorbate reductase (MDHAR) assay kits were purchased from Cohesion Biosciences, London, UK. A glutathione-S-transferase (GST) assay kit was bought from Biorbyt, Cambridge, UK. A glutathione peroxidase (GPx) assay kit was obtained from Cayman Chemicals, Ann Arbor, MI, USA. Ferric-reducing antioxidant power (FRAP) was sourced from Cell Biolabs, San Diego, CA, USA. A 2,2-Diphenyl-1-picrylhydrazyl (DPPH) or Trolox equivalent antioxidant capacity (TEAC) assay kit was obtained from Bioquochem, Ovideo, Spain. MTS CellTiter 96^®^ AQueous One Solution Cell Proliferation reagent was purchased from Promega, Madison, WI, USA. H2DCFDA (2′,7′-Dichlorodihydrofluorescein diacetate) was purchased from Canvax, Cordoba, Spain. Absorbances for all assays were read using a Multiskan FC (Thermofisher Scientific, Dublin, Ireland) or Varioskan Lux (Thermo Scientific, Dublin, Ireland) microplate reader.

### 3.2. Solarplast^®^ and Frozen Spinach Sample Preparation

To obtain plant extracts, 250 mg of Solarplast^®^ and frozen spinach were finely ground to a powdered form with two additions of liquid nitrogen (approx. 200 mL) in a pre-cooled unglazed porcelain 90 mm diameter mortar and pestle (Scientific Laboratory Supplies, Dublin, Ireland). One hundred milligrams of finely ground samples were suspended in 2 mL ice cold PBS (pH 7.4), or with appropriate assay buffers for the CAT, GST and MDHAR assays available in the enzyme kits as indicated by the manufacturer’s instructions. The samples were vortexed vigorously for 1 min and centrifuged at 9800× *g* for 15 min at 4 °C (MPW-352R, Medical Supply Company, Dublin, Ireland), and the supernatant filtered through a 0.45µm filter syringe (Scientific Laboratory Supplies, Ireland) into a fresh tube producing an extract at 50 mg/mL for each sample. The extracted samples were freshly prepared and maintained at 4 °C for each assay. 

### 3.3. Enzymatic and Small Molecules Antioxidant Capacity in Solarplast^®^ and Frozen Spinach

Antioxidant activity of Solarplast^®^ and frozen spinach was measured from extracted supernatants using TOC, FRAP, DPPH-TEAC, SOD, CAT, POD, GST, MDHAR and GPx kits following detailed manufacturer’s instructions. 

### 3.4. Total Antioxidant Assay

Total antioxidant activity was assessed from freshly extracted supernatants of Solarplast^®^ and frozen spinach using the total antioxidant capacity (TOC), ferric-reducing antioxidant power (FRAP) and 2,2-Diphenyl-1-picrylhydrazyl (DPPH-TEAC) assays. 

In TOC assay, 100 µL of Cu^2+^ working reagent is added to 20µL of the sample and incubated at room temperature for 90 min. Cu^2+^ is reduced to Cu^+^ ions and it chelates with a colorimetric probe, giving a broad absorbance peak at ~570 nm. The chelation is proportional to the total antioxidant capacity. The concentration of Trolox equivalent (nmole/µL) was calculated from a Trolox equivalent standard curve and the given formula from the manufacturer’s manual. The concentration was converted from nmole/µL to µmol/min/g to obtain U/g.

For the quantitative FRAP assay, 100 µL of iron-colorimetric probe reaction mix was added to 100 µL of the sample and was incubated at room temperature for 10 min. The presence of antioxidants in the sample reduces ferric ions (Fe^3+^) to ferrous (Fe^2+^). This reduction is coupled with the iron-colorimetric probe complex and develops a dark blue colour which was measured at 570 nm. 

In DPPH assay, 200 µL of DPPH reaction agent was added to 20 µL of the sample and mixed well. The antioxidant compounds found in a sample transfer electrons to DPPH^+^ which discolourises from purple to yellow. This discoloration reaction is proportional to the antioxidant activity in the sample and was measured at 520 nm. DPPH inhibition (%) was converted to TEAC (µM) according to the manufacturer’s instructions to obtain antioxidant activity in U/g.

### 3.5. Superoxide Dismutase (SOD)

The assay kit uses tetrazolium salt (WST-1) to produce a formazan dye upon reduction with a superoxide anion. Superoxide anion is proportional to the absorbance, and SOD activity was quantified by measuring the decrease in colour development at 450 nm. Two hundred microlitres of WST working solution was added to 20 µL of enzyme working solution and 20 µL of the sample. The reactions were incubated at 37 °C for 20 min and the standard curve was used to convert the SOD concentrations in U/mL to U/g. 

### 3.6. Catalase (CAT)

The assay initiates enzymatic hydrolysis of hydrogen peroxide by catalase. Twenty microlitres of sample were mixed with 80 µL of the substrate solution and incubated for 3 min at room temperature. After incubation, 100 µL of the dye reagent was added to the reaction mix and the absorbance was measured at 405 nm. The concentration of catalase in U/g was calculated using the formula specified in the manufacturer’s manual. 

### 3.7. Peroxidase (POD)

Peroxidase reaction mix was prepared by combining 46 µL of the assay buffer, 2 µL fluorescent peroxidase substrate and 2 µL of 12.5 mM hydrogen peroxidase substrate. Fifty microlitres of the sample was added to 50 µL of the reaction mix. A colorimetric product was produced when peroxidase in the sample catalysed the reaction between hydrogen peroxide and the probe. All reactions were incubated at 37 °C for 120 min prior to measuring absorbances at 570 nm. Peroxidase activity was calculated in nmole/min/mL and U/g using the given formula from the manufacturer’s instructions. 

### 3.8. Glutathione-S-Transferase (GST)

Twenty microlitres of the sample were added to 160 µL of the reaction buffer, 10 µL of substrate I and 10 µL of the substrate II from the assay kit. GST in the sample catalyses reaction between GSH and the GST substrate, CDNB (1-chloro-2,4-dinitrobenzene) to form dinitrophenyl thioether which was measured at 340 nm at an initial time point and after incubating at room temperature for 2 min. The concentration of GST in U/g was calculated using the given formula in the manufacturer’s manual. 

### 3.9. Monodehydroascorbate Reductase (MDHAR)

Ten microlitres of the sample were added to 170 µL of reaction buffer, 10 µL enzyme and 10 µL of the substrate available in the assay kit. MDHAR catalyses NADH and the enzymatic activity was measured at 340 nm at the initial time point and after 2 min of incubation at room temperature. The concentration of MDHAR was calculated from the standard curve in µmol/L and then converted to U/g. 

### 3.10. Glutathione Peroxidase (GPx)

A reaction mixture was prepared by adding 20 µL of the sample to 50 µL of the assay buffer, 50 µL of the co-substrate mixture and 50 µL of NADPH. The reaction was initiated by adding 20 µL of the cumene hydroperoxide and mixing well. The GPx activity was indirectly measured by a coupled reaction with glutathione reductase. The final oxidation reaction from NADPH to NADP+ was accompanied by a decrease in absorbance at 340 nm. The concentration of GPx in U/g was calculated using the given formula in the manufacturer’s manual.

### 3.11. Cell Culture Conditions

HT-29 (ATCC HTB-38™; Sigma-Aldrich, Wicklow, Ireland) cells were routinely maintained in DMEM cell culture media supplemented with 10% fetal bovine serum, 1% non-essential amino acids solution, 100 U/mL penicillin, 100 μg/mL streptomycin and 1% L-Glutamine. Cells were incubated at 37 °C under 5% CO_2_ atmosphere. 

HepG2 (ATCC HB-8065™; American Type Culture Collection (ATCC), Middlesex, UK) cells were routinely maintained in EMEM cell culture media supplemented with 10% fetal bovine serum, 1% non-essential amino acids solution, 100 U/mL penicillin, 100 μg/mL streptomycin and 1% L-Glutamine. Cells were incubated at 37 °C under 5% CO_2_ atmosphere. 

### 3.12. Solarplast^®^ Extract Cytotoxicity in HT-29 and HepG2 Cell Lines

The effect of Solarplast^®^ on the viability of the HT-29 and HepG2 cell lines was determined using XTT assay. HT-29 and HepG2 cells were seeded at a density of 5 × 10^4^ and 6.5 × 10^5^ cells per well in 96 well plates, respectively. The plates were incubated at 37 °C, 5% CO_2_ for 24 h to allow for cell attachment. The following day, cells were treated with Solarplast^®^ at the following concentrations: 5 mg/mL, 2 mg/mL and 1 mg/mL. All samples including DMSO (10%) were prepared in the corresponding cell culture media. DMSO was used as a positive control for cell cytotoxicity. The 96 well plates were then returned to the incubator and incubated at 37 °C, 5% CO_2_ for 24 h. The following day, the media containing the treatment was removed and cells were washed with PBS. One hundred microlitres of cell culture media and 50 μL of XTT reagent were added to each treated well. The 96 well plate was incubated at 37 °C, 5% CO_2_ for 2 h. Absorbance reading at 450 nm was recorded using a Multiskan FC plate reader.

### 3.13. Solarplast^®^ Antioxidant Capacity in HT-29 and HepG2 Cell Lines 

Antioxidant assays were performed using fluorescent dye—DCF-DA. HT-29 and HepG2 cells were seeded in 100 μL complete media at a density of 5 × 10^4^ and 6.5 × 10^4^ cells per well in 96 well plates, respectively. Cells were allowed to adhere overnight. The following day cells were washed three times with PBS prior to treatment. The cells were then treated with Solarplast^®^ at the following concentrations: 5 mg/mL, 2 mg/mL and 1 mg/mL. Ascorbic acid (vitamin C) at 1 mg/mL concentration was used as a positive control for the antioxidant activity. All samples including positive control were prepared in the corresponding cell culture media. The 96 well plates were then returned to the incubator and incubated at 37 °C, 5% CO_2_ for 24 h. The following day, the medium containing the treatment was removed and cells were washed with PBS. The cells were then stained with fluorescent dye at 60 μM DCF-DA concentration in serum-free cell culture media for 50 min. The medium containing the stain was removed and cells were washed with PBS twice prior to addition of insults. Insults at a concentration of 30 mM acetaminophen, 2 mM hydrogen peroxide (H_2_O_2_) and 6% ethanol were prepared in the corresponding serum-free cell culture media. The cells were exposed to the insult for 90 min. Fluorescence was measured using a Varioskan lux 485 emission and 538 nm excitation filter set.

### 3.14. Statistical Analysis 

Data and graphs were prepared and analysed using GraphPad prims 9.1.1 (GraphPad Software, San Diego, CA, USA). All datasets were analysed for normality distribution using the D’Angostino–Pearson test. For pair comparisons, statistical differences between groups were analysed using the unpaired *t*-test or Mann–Whitney U test depending on results of normality tests. For multiple comparisons, One-Way ANOVA with Dunnett post-hoc was used for samples with normal distribution, whereas Kruskal–Wallis with Dunn’s post-hoc was performed when datasets did not follow normal distribution.

## 4. Discussion

Reactive oxygen species including intermediates (peroxides), like superoxide radicals (O_2_^•−^), hydrogen peroxide (H_2_O_2_), hydroxyl radicals (^•^OH) and singlet oxygen (^1^O_2_), are constantly being generated in our cells through metabolic activity [27]. The overproduction of such molecules and radicals can cause oxidative damage to lipids, proteins and DNA [3,4], contributes to the ageing process, and can lead to cell death and a host of disorders [5]. Our bodies regulate oxidative stress through endogenous and dietary antioxidant molecules that can prevent the oxidation of a substrate at low concentrations [1]. 

Epidemiological observations and studies support the notion that consumption of antioxidant-rich food is important to reduce the risk of a number of disorders including, but not limited to, diabetes, obesity and cardiovascular disease in humans [28,29,30,31,32,33,34]. Amongst vegetables, spinach has been shown to contain high concentrations of antioxidant enzymes and molecules compounds in either its raw or powder form [16,35,36,37]. The majority of studies looking at the antioxidant contribution of dietary supplements and food components have assessed the overall antioxidant contribution (total antioxidant capacity), as vegetables and other plant-based products contain many hundreds of compounds with potential antioxidant activity, making it unfeasible to quantify all of these individually due to geographical sourcing, harvesting, storage and processing conditions [14,38]. 

In this study, the antioxidant efficacy of Solarplast^®^, a novel, non-GMO supplement derived from frozen blanched spinach undergoing proprietary processing with specific conditions in the presence of an enzyme formulation was compared with frozen blanched spinach controls using three different validated antioxidant assays (TOC, TEAC and FRAP). These assays are based on different antioxidant pathways and were selected to consider the wide variety and range of action of antioxidant compounds, while the enzymes investigated represent a plethora of enzymatic mechanisms by which antioxidants mediate their effects including the glutathione pathway, and superoxide dismutase, peroxidase, catalase and monodehydroascorbate reductase activity. FRAP and TEAC antioxidant levels of the control frozen spinach were 83.7 ± 2.9 U/g (83.7 mmol/100 g) and 6.7 ± 0.0 U/g (0.6 mmol/100 g), respectively. These values were slightly different to previous reports at 15.98 ± 0.51 mmol/100 g and 4.43 ± 0.11 mmol/100 g, suggesting that processing methods and storage conditions can have a significant impact on final results achieved in this study [39]. Comparatively, Solarplast^®^ activity was 5× and >200× times higher in the FRAP and TEAC assay when compared to frozen spinach (Figure 1). Moreover, Solarplast^®^ Trolox levels (60.3 ± 2 µM/g) were higher than these observed in acerola (36.88 ± 1.54 µM/g), acai (26.02 ± 4.68 µM/g) and camu-camu (33.74 ± 5.49 µM/g) extracts [40]. Altogether, Solarplast^®^ demonstrated superior antioxidant enzymatic activity and greater antioxidant capacity than the frozen spinach (Figure 2). In the formulation process, the plant cell wall of the spinach cells is broken down resulting in concentrated spinach protoplasts instead of whole spinach cells, which may explain the observed superior antioxidant profile. This process serves to concentrate protoplasts containing the peroxisome, chloroplasts, mitochondria and cytoplasm where most antioxidant molecules are synthesised and stored, and removes extracellular components that may have no intrinsic antioxidant activity [41]. Interestingly, the catalase activity of the Solarplast^®^ was less efficacious than the frozen spinach extract, suggesting that the formulation process (removal of the cell walls) may influence this specific activity. Regardless, there was still catalase activity in the Solarplast^®^ preparation. Together, these findings suggest that despite blanching, and the enzymatic treatment and freeze-drying process for Solarplast^®^, the enzymes present in these preparations still demonstrate antioxidant activity, with a superior profile being demonstrated by Solarplast^®^. 

To further investigate the functional efficacy of Solarplast^®^ as an antioxidant, we assessed the protective effect of Solarplast^®^ in well-validated cell models of oxidative stress, using acetaminophen, ethanol and hydrogen peroxide to induce reactive oxygen species in situ and vitamin C as a well-studied and validated positive antioxidant control. There are several studies that have characterised plant extracts for their capacity to prevent oxidative stress damage in different cell lines, including those of large intestine (HT-29) and liver (HepG2). Thai pigmented rice extracts reduced ROS in HT-29 exposed to H_2_O_2_ [42], though the efficacy varied depending on the type of rice used to obtain the extracts. In HepG2, cell extracts of *Salvia officinalis* and *Thymus vulgaris* reduced DNA damage induced after exposure to 300 µM of H_2_O_2_ [43]. In this study, Solarplast^®^ reduced ROS levels in both HT-29 and HepG2 cells exposed to H_2_O_2_ (Figure 4). Ethanol metabolism has been associated with increased oxidative stress, and a number of phytochemicals including beta carotene, betaine, curcumin, ellagic acid, epigallocatechin-3-gallate, ferulic acid, hydroxystilbenes, lutein, morin and meso-zeaxanthin have been studied for their hepatoprotective effects against ethanol-induced hepatotoxicity. To date, numerous mechanisms of hepatoprotection by most phytochemicals have been elucidated, which mainly include increased scavenging of ethanol-derived hydroxyl and hydroxy ethyl radicals by increasing endogenous antioxidants (CAT, SOD, GPX and glutathione) and inhibiting the induction of CYP2E1, involved in the generation of ROS [44,45]. Comparably, in this study, Solarplast^®^ reduced ROS levels in both HT-29 and HepG2 cells exposed to 6% ethanol (Figure 5). However, further investigation is required for better understanding the mechanisms responsible for the protective effects of Solarplast^®^ against alcohol-induced toxicity in these cell lines. Additionally, phytochemicals have been reported to have effects of alleviating acetaminophen-induced liver injury, predominantly by reducing free-radical production, lipid peroxidation, inhibiting the binding of toxins to the hepatocyte cell membrane receptors and reducing the superoxide and peroxynitrite content by their scavenging activity [46]. In addition to hydrogen peroxide and ethanol, 30 mM acetaminophen induced oxidative stress in both HT-29 and HepG2 cells, and this was significantly attenuated by Solarplast^®^ extracts across all concentrations tested (Figure 6). The evidence herein whereby Solarplast^®^ reduced ROS levels in both HT-29 and HepG2 cells exposed to H_2_O_2_, ethanol and acetaminophen combined with its high TOC, TEAC and FRAP antioxidant levels validates that it has antioxidant activity and suggests it may represent a valid dietary means by which to contribute to the control of oxidative stress.

## 5. Conclusions

In this work, preliminary analysis of the total antioxidant activity of Solarplast^®^, a novel, commercially available extract prepared from non-GMO, blanched frozen spinach is investigated using a combination of analytical techniques and in vitro cell-based assays. To our knowledge, this is the first study to assess the antioxidant potential of an enzymatically formulated concentrated spinach protoplast supplement. The results demonstrate a superior antioxidant profile of Solarplast^®^ as compared with frozen spinach leaves and these were comparable with or superior to other plant extracts reported previously [38]. Chemical identities and quantities of these antioxidant components in Solarplast^®^ will be investigated and compared to those in fresh, frozen and other spinach extracts obtained by different extraction methods in the future. Furthermore, the biological activity of Solarplast^®^ was characterised using human in vitro cell models (HepG2 and HT29), where Solarplast^®^ acted on ROS by lowering the oxidative damage induced by three known oxidants. Overall, the high total antioxidant activity and ROS-reducing abilities of Solarplast^®^ suggest that this spinach extract supplement may represent an effective method to contribute to lowering oxidative stress through dietary means. 

## Data Availability

Results of all analyses are included in this published article. The datasets generated and/or analysed during the current study are available from the corresponding authors on reasonable request.
